# Normal tissue and tumor microenvironment adaptations to aerobic exercise enhance doxorubicin anti-tumor efficacy and ameliorate its cardiotoxicity in retired breeder mice

**DOI:** 10.18632/oncotarget.28057

**Published:** 2021-08-31

**Authors:** Zachary R. Wakefield, Mai Tanaka, Christine Pampo, Sharon Lepler, Lori Rice, Joy Guingab-Cagmat, Timothy J. Garrett, Dietmar W. Siemann

**Affiliations:** ^1^Department of Radiation Oncology, College of Medicine, University of Florida, Gainesville, FL 32610, USA; ^2^Department of Pathology, Immunology, and Laboratory Medicine, College of Medicine, University of Florida, Gainesville, FL 32610, USA

**Keywords:** aerobic exercise, breast cancer, hypoxia, doxorubicin, cardiotoxicity

## Abstract

Aerobic exercise is receiving increased recognition in oncology for its multiple purported benefits. Exercise is known to induce physiologic adaptations that improve patient quality-of-life parameters as well as all-cause mortality. There also is a growing body of evidence that exercise may directly alter the tumor microenvironment to influence tumor growth, metastasis, and response to anticancer therapies. Furthermore, the physiologic adaptations to exercise in normal tissues may protect against treatment-associated toxicity and allow for greater treatment tolerance. However, the exercise prescription required to induce these beneficial tumor-related outcomes remains unclear. This study characterized the aerobic adaptations to voluntary wheel running in normal tissues and the tumor microenvironment. Female, retired breeder BALB/c mice and syngeneic breast adenocarcinoma cells were utilized in primary tumor and metastasis models. Aerobic exercise was found to induce numerous adaptations across various tissues in these mice, although primary tumor growth and metastasis were largely unaffected. However, intratumoral hypoxia and global metabolism were altered in the tumors of exercising hosts relative to non-wheel running controls. Doxorubicin chemotherapy also was found to be more efficacious at delaying tumor growth with adjuvant aerobic exercise. Additionally, doxorubicin-induced cardiac toxicity was ameliorated in exercising hosts relative to non-wheel running controls. Taken together, these data suggest that the normal tissue and tumor microenvironment adaptations to aerobic exercise can improve doxorubicin efficacy while simultaneously limiting its toxicity.

## INTRODUCTION

Cancer presents a major public health concern both in the United States and worldwide. In females, breast cancer is the most commonly diagnosed malignancy and is responsible for the second most deaths across all cancer types [1]. Triple-negative breast cancer, characterized by the lack of expression of estrogen receptor, progesterone receptor, and human epidermal growth factor 2, is a particularly aggressive subtype with poor outcomes [2]. As such, there is a pressing need for improved modalities for the management and treatment of this disease [3]. Aerobic exercise is intriguing as a potential adjuvant therapy for breast cancer. When implemented either during or after treatment, exercise has been shown to result in reduced adverse events, and to improve quality-of-life and aerobic respiratory fitness in cancer patients [4–6]. In addition, clinical trials have shown that exercise can decrease risk of breast cancer recurrence [7, 8]. Consequently, exercise recommendations are being adopted by major national and international cancer organizations [9–11].

Still, the therapeutic potential of including exercise in clinical cancer management is far from fully realized. Emerging clinical data suggest that in addition to the known physical and psychological benefits, aerobic exercise may also improve cancer treatment efficacy and limit associated toxicity [12]. Although the underlying mechanisms responsible for these effects remain largely unknown, evidence points to exercise’s impact on host physiology and the tumor microenvironment being involved. Preclinical data are limited, but studies have shown that key components of the tumor microenvironment, including hypoxia, immune cell infiltration, and metabolism, may be impacted by exercise [13]. Consequently, these exercise effects may have the potential to affect tumor growth, tumor cell dissemination, and tumor response to anticancer therapies. Furthermore, normal tissue adaptations to aerobic exercise may protect against the toxicity of anticancer therapy. This may be of particular relevance for breast cancer survivors in whom cardiovascular disease is increasingly prevalent [14–16]. Various chemotherapies are known to be cardiotoxic [17] and doxorubicin, a highly prescribed chemotherapeutic agent for the treatment of breast cancer [18], has cumulative and dose-dependent cardiotoxic effects. Importantly, preclinical and clinical data suggest that exercise implemented before, during, and after doxorubicin administration may limit the extent of cardiac toxicity [19–22].

Despite generally positive findings in regard to exercise and cancer outcomes, it remains unclear how to properly prescribe exercise. A recent review of preclinical exercise oncology found that methodologic heterogeneity and incomplete characterization of the exercise made it difficult to link the exercise prescription to the tumor effect [23]. Hence, improved characterization of exercise interventions and responses in preclinical studies will better guide clinical study design. Here, we employed voluntary wheel running in orthotopic, syngeneic models of breast cancer in aged mice. The goal of this study was to characterize the exercise prescription by evaluating the aerobic adaptations in both the normal tissue and the tumor microenvironment. Moreover, doxorubicin was used to assess the adjuvant effects of aerobic exercise on chemotherapy efficacy and toxicity.

## RESULTS

### Voluntary wheel running induces aerobic adaptations

**Figure 1:**
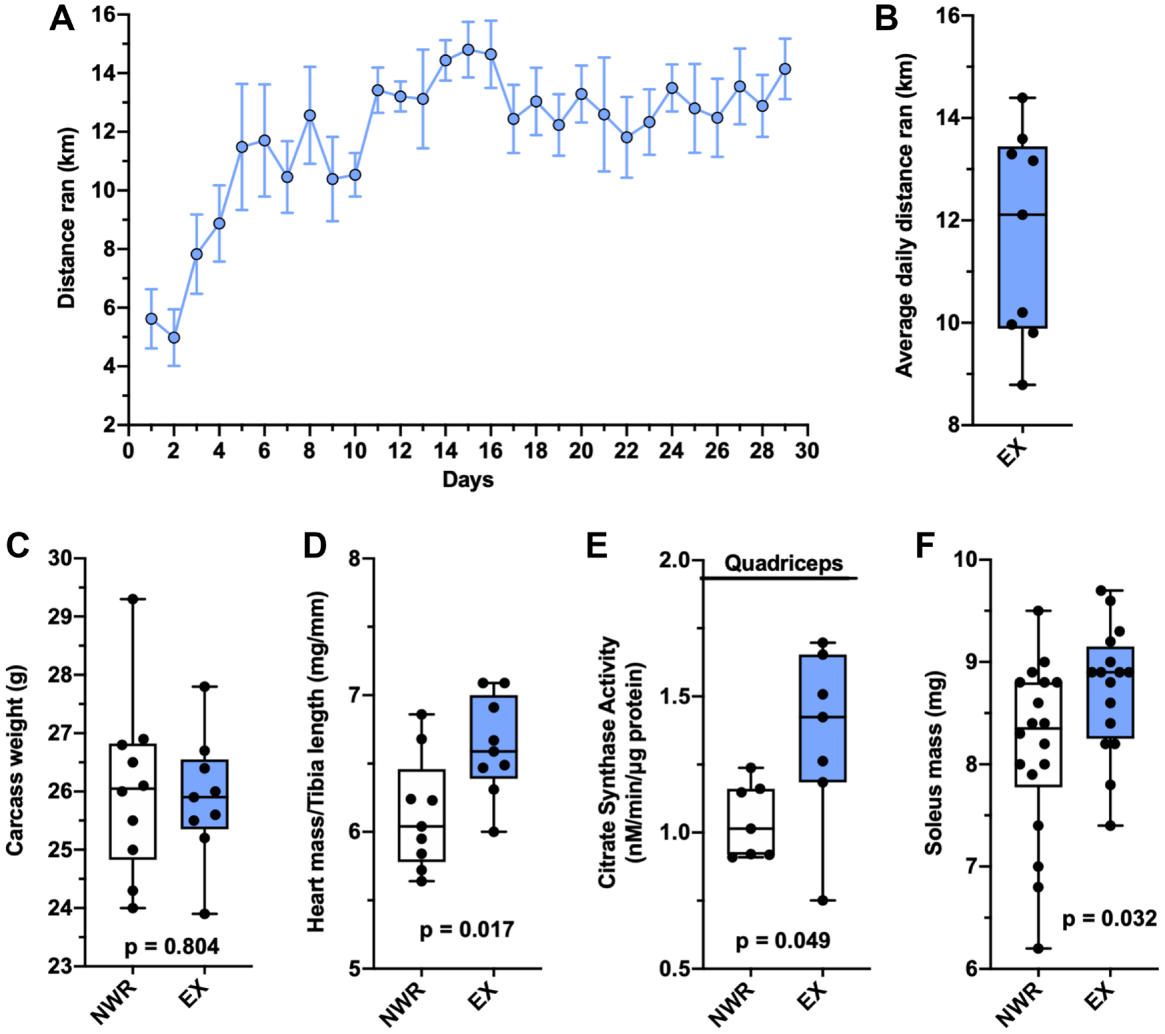
Voluntary wheel running induces aerobic adaptations. Average distance ran per 24-hour period ± SEM in female, retired breeder BALB/c mice with EMT6 intraductal tumors (**A**). Individual values for average daily distance ran throughout the study (**B**). Carcass mass (g; calculated as bodyweight minus tumor mass; (**C**). Heart mass to tibia length ratio (mg/mm; (**D**). Activity rates of the citrate synthase enzyme from quadriceps homogenates, *n* = 7 per group (nM/min/μg protein; (**E**). Soleus mass (mg; (**F**). Welch’s *t*-test was used to statistically compare means between groups, *p* values denoted in panels, study sample size: NWR *n* = 10; EX *n* = 9.

Retired breeder BALB/c mice were intraductally inoculated with syngeneic EMT6 cells and randomized into non-wheel running (NWR) and exercising (EX) groups. Everyday wheel access was chosen as the exercise modality as exercising mice in this group displayed greater exercise adaptations than mice given limited wheel access (Supplementary Figure 1). The daily running behavior steadily increased over the first week these retired breeder mice were given access to the running wheels, and then their running behavior plateaued at around 12 km per 24-hour period (Figure 1A). The average daily distance ran per mouse ranged from roughly 9 km/day to 14 km/day (Figure 1B). At endpoint, these mice displayed aerobic adaptations across multiple tissues consistent with aerobic exercise training. Bodyweight was not affected (Figure 1C), however cardiac hypertrophy was observed in the heart mass to tibia length ratio (Figure 1D). In the quadriceps muscle, the enzymatic activity of citrate synthase was elevated, indicative of increased mitochondrial density (Figure 1E). Hypertrophy of the soleus muscle was also observed (Figure 1F).

### Aerobic exercise ameliorates intratumoral hypoxia

**Figure 2:**
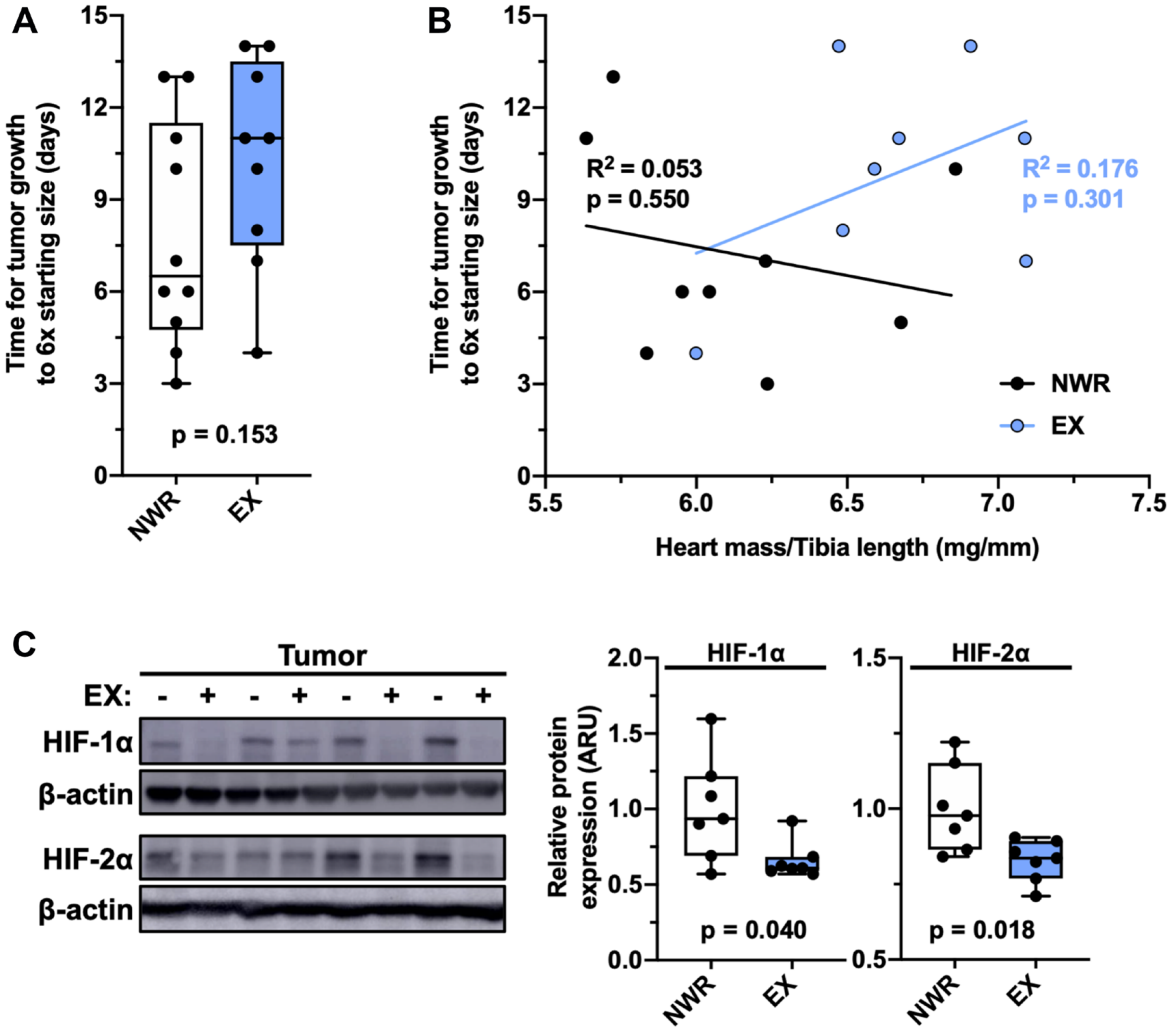
Aerobic exercise ameliorates intratumoral hypoxia. Time (days) for EMT6 tumor growth from 200–1200 mm^3^ (**A**). Linear regression of individual normalized heart mass and tumor growth time, Pearson’s R^2^ values denoted in panel (**B**). Representative immunoblot for HIF-1α, HIF-2α, and β-actin and relative quantifications, *n* = 7 per group (**C**). Welch’s *t*-test was used to statistically compare means between groups, *p* values denoted in panels, study sample size: NWR *n* = 10; EX *n* = 9.

Intraductal EMT6 tumor growth was not significantly affected by aerobic exercise when comparing the time, in days, it took for these tumors to grow 6 times starting size (defined as > 200 mm^3^; Figure 2A). Individual tumor growth positively correlated with normalized heart mass in exercising mice but not non-wheel running mice (Figure 2B), suggesting that the tumors grew slower in the hosts which displayed greater exercise adaptation. Intratumoral hypoxia was evaluated by immunoblotting for HIF-1α and HIF-2α expression, endogenous indicators of acute and chronic hypoxia, respectively. Exercising mice displayed reduced expression of both markers (Figure 2C).

### Aerobic exercise influences intratumoral metabolism

**Figure 3:**
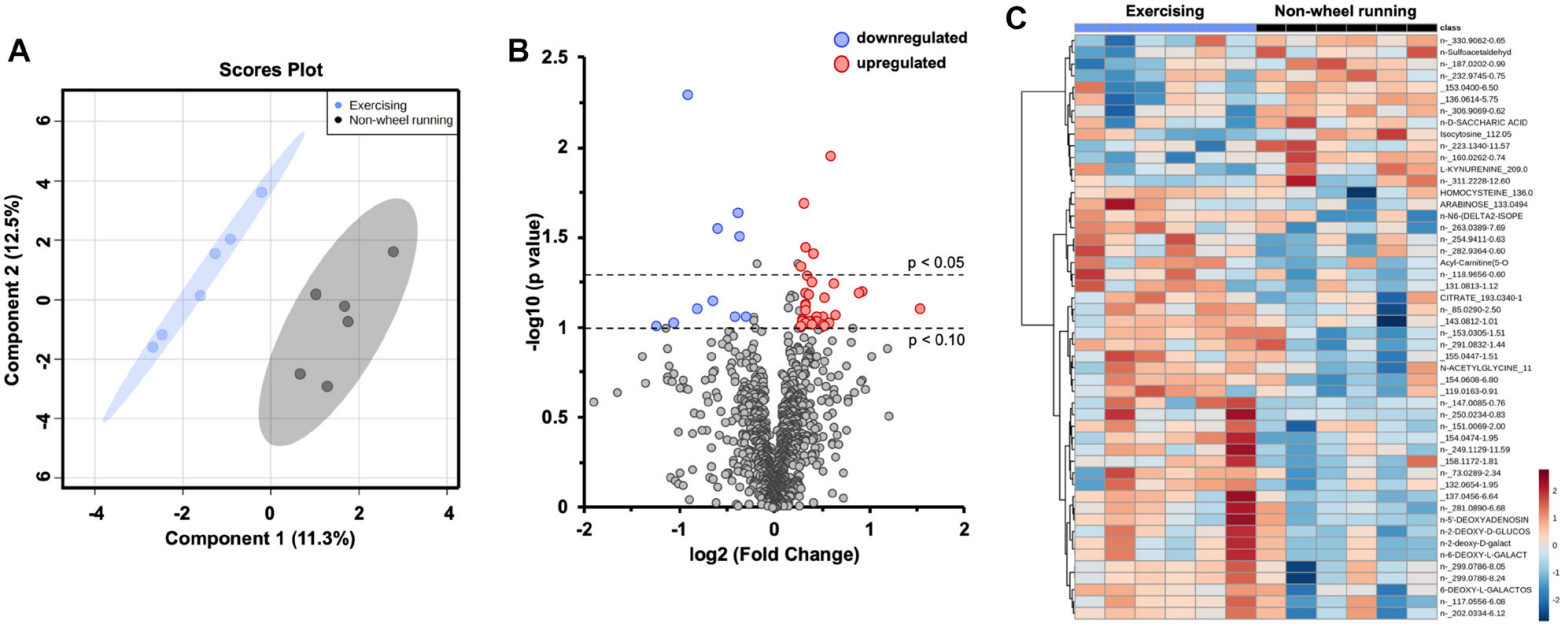
Aerobic exercise influences intratumoral metabolism. Global metabolomics profiling of EMT6 intraductal tumors from non-wheel running and exercising hosts. Sparse partial least squares discriminant analysis (sPLSDA) of 5 components and 20 variables per component (**A**). Volcano plot of individual metabolites, fold change threshold of 20% and *p* values of 0.05 and 0.10 denoted with black, dotted lines (**B**). Hierarchical clustering was performed on the top 50 differential metabolites from *t*-test (**C**). Study sample size: NWR *n* = 6; EX *n* = 6.

To investigate the impact of exercise on tumor metabolism, global metabolomics profiling was performed on EMT6 tumors via mass spectrometry. Sparse PLS discriminant analysis (sPLSDA) revealed differential metabolomic profiles between non-wheel running and exercising groups (Figure 3A). At a fold change threshold of 20%, 10 metabolites were downregulated, and 32 metabolites upregulated (Figure 3B). Hierarchical clustering of the top 50 metabolites in non-wheel running and exercising groups was performed (Figure 3C). The complete list of metabolite fold change can be found in Supplementary Table 1.

### Pulmonary metastasis is not affected by aerobic exercise

**Figure 4:**
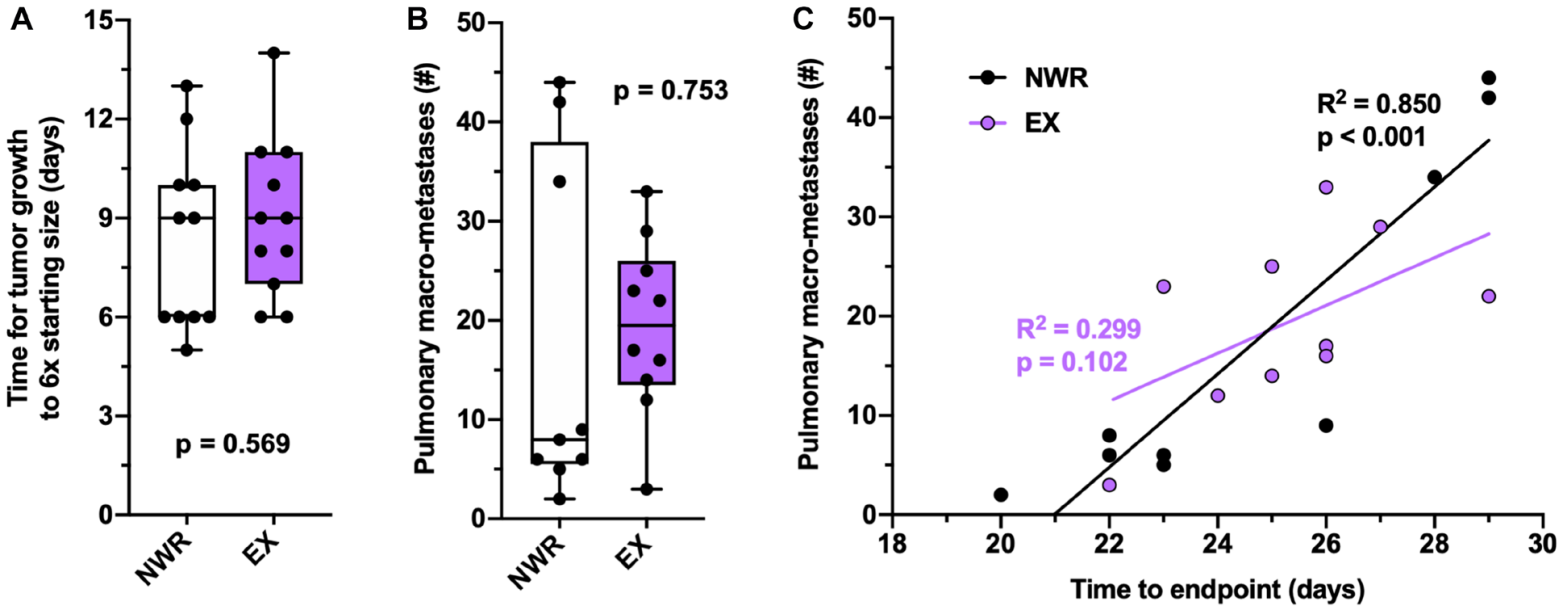
Pulmonary metastasis is not affected by aerobic exercise. Time (days) for 4T1 tumor growth from 200–1200 mm^3^ (**A**). Pulmonary macro-metastases counted under microscope (**B**). Correlation between number of pulmonary macro-metastases to time (days) to endpoint, Pearson’s R^2^ values denoted in panel (**C**). Welch’s *t*-test was used to statistically compare means between groups, *p* values denoted in panels, study sample size: NWR *n* = 11; EX *n* = 11.

Pulmonary metastasis was not observed in this EMT6 model, thus the aggressive 4T1 breast cancer cell line was employed to evaluate the effect of exercise on spontaneous metastasis. Intraductal 4T1 tumor growth was unaffected by exercise (Figure 4A) although the 4T1 tumor model affected running behavior in the plateau phase, decreasing the distances ran as these tumors grew to endpoint (Supplementary Figure 2A). Despite this drop-off in running behavior, these mice still displayed aerobic adaptations in normalized heart mass and soleus mass relative to non-wheel running controls (Supplementary Figure 2B–2C). Pulmonary macro-metastases were counted at endpoint, and exercise did not significantly affect the number of pulmonary metastasis (Figure 4B). Both non-wheel running and exercising groups similarly correlated time to endpoint with pulmonary metastasis (Figure 4C).

### Aerobic exercise increases the anti-tumor efficacy of doxorubicin chemotherapy

**Figure 5:**
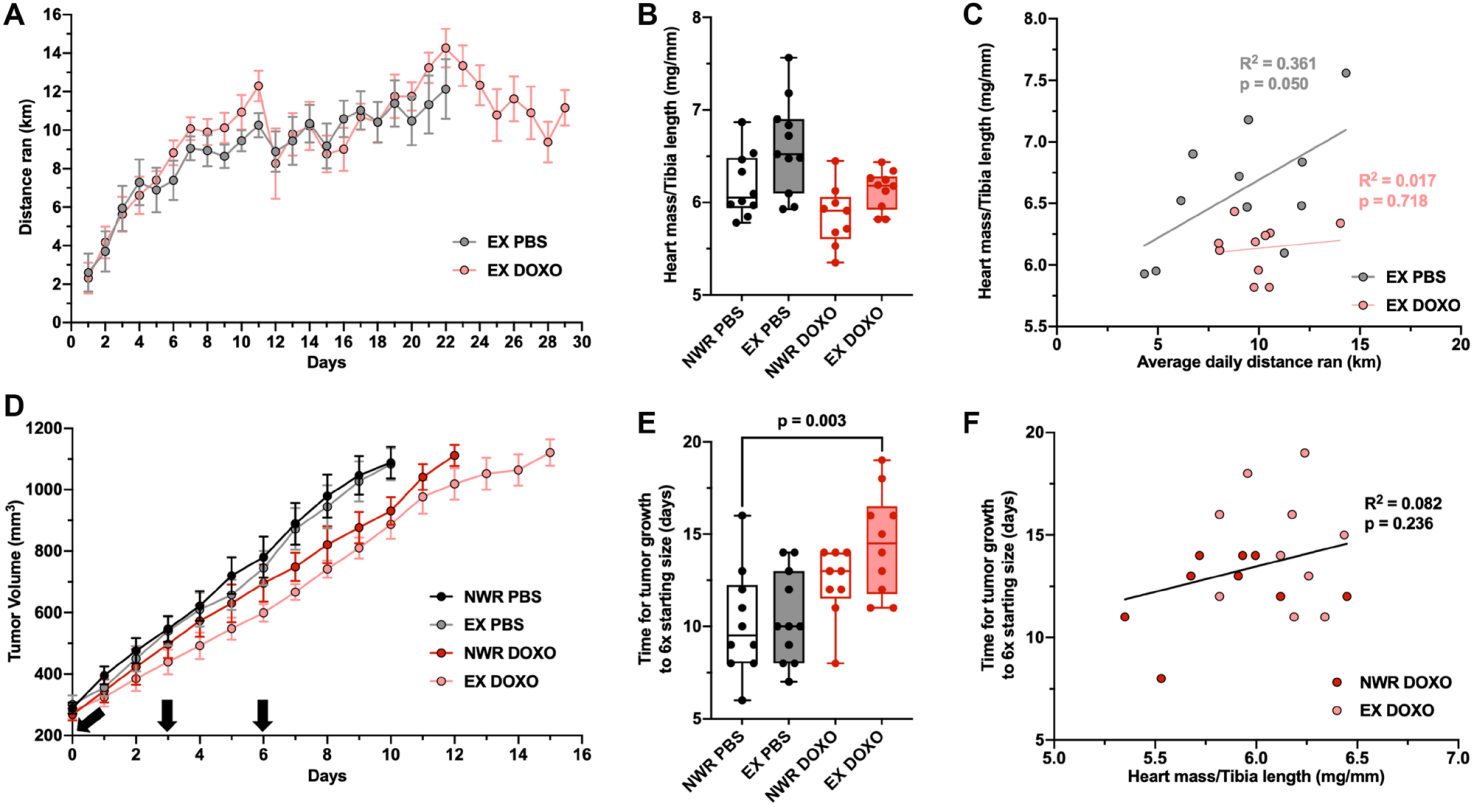
Aerobic exercise increases the anti-tumor efficacy of doxorubicin chemotherapy. Average distance ran per 24-hour period ± SEM in female, retired breeder BALB/c mice with EMT6 intraductal tumors (**A**). Heart mass to tibia length ratio (mg/mm; (**B**). Linear regression of individual average daily distance ran and normalized heart mass, Pearson’s R^2^ values denoted in panel (**C**). Average EMT6 tumor volumes (mm^3^) from 200–1200 mm^3^ (**D**). Black arrows denote injections of PBS or DOXO on days 0, 3, and 6 starting from tumor volume ≥ 200 mm^3^ (total dose 11 mg/kg). Time (days) for EMT6 tumor growth from 200–1200 mm^3^ (**E**). Linear regression of individual normalized heart mass and tumor growth time, Pearson’s R^2^ values denoted in panel (**F**). Two-way ANOVA was used to statistically compare means between groups with Tukey’s post-hoc analysis, detailed analysis in Supplementary Figure 3, *p* values denoted in panels (p^*^ ≤ 0.05), study sample size: NWR PBS *n* = 10; EX PBS *n* = 11; NWR DOXO *n* = 9; EX DOXO *n* = 10.

To evaluate aerobic exercise as an adjuvant therapy to doxorubicin treatment, the intraductal EMT6 model was utilized as tumor growth did not affect running behavior, unlike 4T1 tumors. Upon tumor volume ≥ 200 mm^3^ (which took on average 15 ± 4 days post-tumor inoculation), mice from both non-wheel running and exercising groups were further randomized into PBS or doxorubicin (DOXO) groups. Treatment regimen consisted of 3 injections spaced over 7 days for a total dose of 11 mg/kg. Doxorubicin treatment did not influence running behavior relative to PBS control (Figure 5A). However, doxorubicin treatment did blunt the cardiac hypertrophy in exercising mice (Figure 5B), so that the average daily distance ran did not correlate with normalized heart mass, unlike in PBS control exercising mice (Figure 5C). Neither exercise nor doxorubicin alone significantly altered tumor growth (Figure 5D). However, the combination of the two did significantly delay tumor growth from non-wheel running PBS control tumors, indicating that exercise increased the anti-tumor efficacy of doxorubicin chemotherapy (Figure 5E). No correlation was observed between normalized heart mass and tumor growth rates in doxorubicin-treated mice via linear regression (Figure 5F).

### Aerobic exercise ameliorates doxorubicin-induced cardiotoxicity

**Figure 6:**
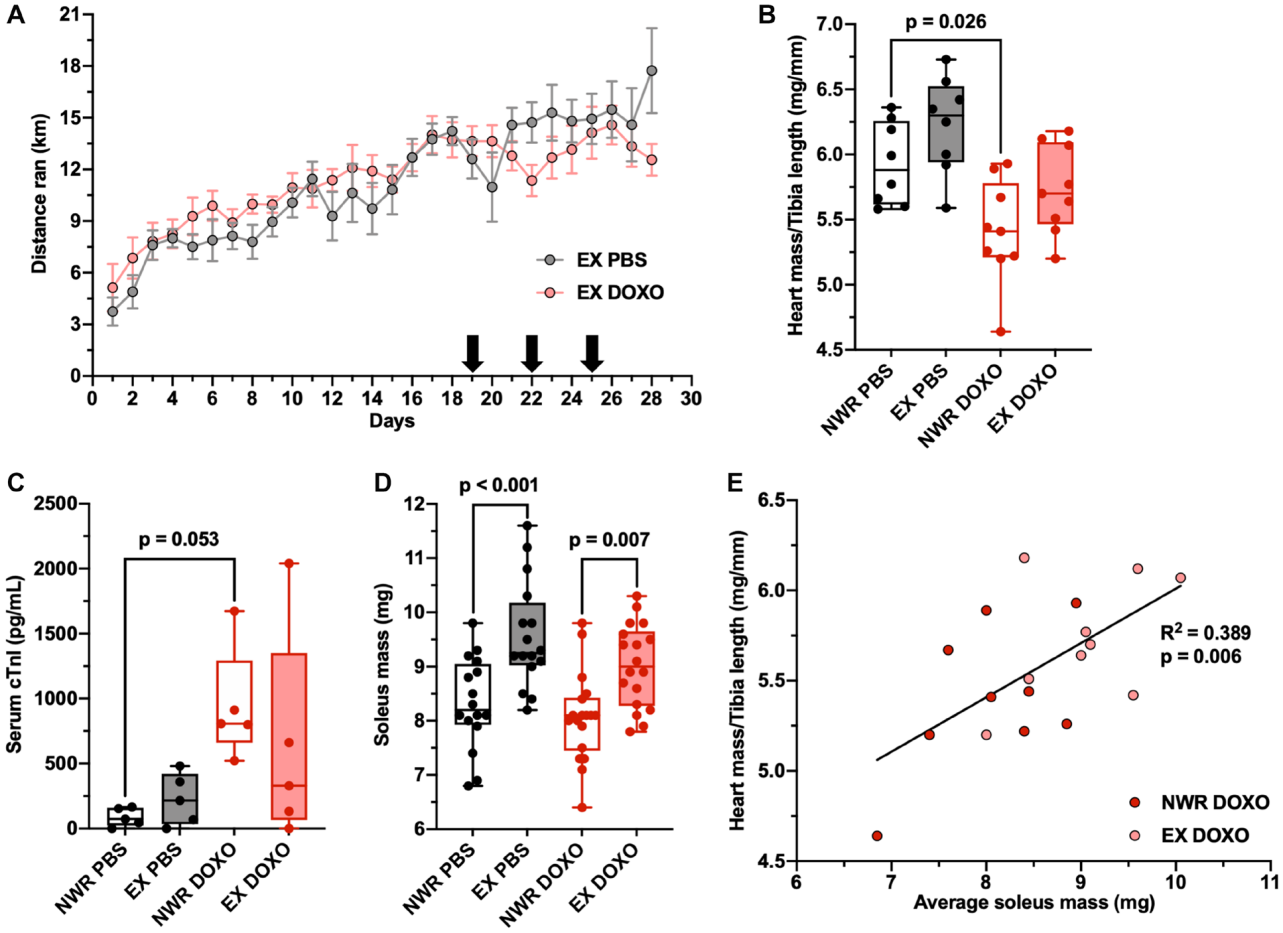
Aerobic exercise ameliorates doxorubicin-induced cardiotoxicity. Average distance ran per 24-hour period ± SEM in female, retired breeder BALB/c mice (**A**). Black arrows denote injections of PBS or DOXO on days 19, 22, and 25 after initial randomization (total dose 18 mg/kg). Heart mass to tibia length ratio (mg/mm; (**B**)). Circulating levels of cardiac troponin I, *n* = 5 per group (pg/mL; (**C**)). Soleus mass (mg; (**D**)). Linear regression of individual average soleus mass and normalized heart mass, Pearson’s R^2^ values denoted in panel (**E**). Two-way ANOVA was used to statistically compare means between groups with Tukey’s post-hoc analysis, detailed analysis in Supplementary Figure 4, *p* values denoted in panels (p^*^ ≤ 0.05), study sample size: NWR PBS *n* = 8; EX PBS *n* = 8; NWR DOXO *n* = 9; EX DOXO *n* = 9.

To assess the potential for aerobic exercise to protect against doxorubicin-induced cardiotoxicity, non-tumor bearing mice were employed and the endpoint was standardized to 72 hours after the final treatment. As a total dose of 11 mg/kg did not induce significant cardiac atrophy (Figure 5B), a higher total dose of 18 mg/kg was evaluated (administered with same dosing schedule). This higher dose of doxorubicin also did not affect running behavior (Figure 6A) but resulted in significant cardiac atrophy relative to non-wheel running PBS controls (Figure 6B). Importantly, aerobic exercise ameliorated this effect. In addition, our results showed that aerobic exercise induced a slight elevation in serum troponin I levels; a commonly observed effect and not believed to be pathological [24]. Doxorubicin treatment, though, did cause a significant increase in serum troponin I levels, and outside of one outlier, exercise blunted this effect (Figure 6C). This marker is indicative of cardiomyocyte damage, as this protein is unique to the myocardium [25]. Exercising mice showed hypertrophy of the soleus muscle regardless of PBS or doxorubicin treatment (Figure 6D). The average soleus mass of individual mice strongly correlated with normalized heart mass after doxorubicin treatment (Figure 6E), indicative of dose-dependent cardioprotective effects of exercise.

## DISCUSSION

Our present study sought to establish the physiologic adaptations to aerobic exercise in both normal tissue and the tumor microenvironment, and how these adaptations influenced breast cancer growth, metastasis, and response to chemotherapy. Voluntary wheel running was found to enhance the anti-tumor action of doxorubicin while simultaneously blunting its cardiac atrophic effects.

There are conflicting preclinical reports surrounding exercise effects on cancer outcomes [26, 27]. One potential contributing factor to this heterogeneity is that studies can employ animals of different species or age with different exercise prescriptions conducted in a variety of tumor models. These variables can affect the exercise capacity and response in the animals [28], thus any potential exercise effect on cancer outcomes will similarly be influenced. Clearly, it is important to more fully characterize these preclinical exercise interventions and responses so as to facilitate inter-study comparison [23]. We characterized voluntary wheel running in retired breeder mice by individually tracking daily running behavior and assessing tissue morphology and enzymatic activity at endpoint. Cardiac and soleus hypertrophy, and increased mitochondrial density in quadriceps muscle indicated that moderate aerobic training was achieved by free wheel running [29]. These markers of exercise adaptation correlated with distance ran, and thus were further used to correlate various tumor-related outcomes to understand the exercise prescription necessary to enact beneficial effects.

Intratumoral hypoxia was reduced by exercise, as evidenced by decreased expression of the endogenous markers HIF-1α and HIF-2α. Hypoxia is caused by the rapid proliferation of neoplastic cells exceeding capillary growth and resultant abnormal angiogenesis [30]. Prior studies, including those from our lab, have postulated that aerobic exercise may ameliorate intratumoral hypoxia by increasing blood flow during exercise, and/or by tumor vasculature normalization [31–33]. Since hypoxia can modulate many aspects of the tumor microenvironment, including overall metabolism [34, 35] and response to chemotherapy [36], exercise might significantly impact therapeutic outcomes. Indeed, metabolomics profiling revealed differences in exercising versus non-wheel running tumors. Pathway analysis was unable to be performed as roughly 70% of the top 50 differentially regulated metabolites were unknown species. However, various citrate species all trended to be upregulated in exercising tumors (Supplementary Table 1), suggestive of increased flux through the citric acid cycle. This would be expected in the better oxygenated tumors of exercising hosts as HIFα expression promotes aerobic glycolysis [37], although lactate levels were not found to be significantly altered in this study. These observations are consistent with previously published metabolomics profiling [38, 39]. Further study is warranted to examine how metabolites differentially regulated by exercise may impact characteristics of neoplasia.

An important observation in the present study was that doxorubicin tumor treatment was more efficacious when combined with exercise compared to non-wheel running control mice, although it should be noted that there was no statistical difference relative to non-wheel running, doxorubicin-treated mice. This suggests that the improved oxygenation of the exercising tumors resulted in an additive effect to improve chemotherapy response. However, it remains unclear whether this effect of exercise was due to altered agent pharmacokinetics or an altered pharmacodynamic response of the cancer cells. Since doxorubicin is known to poorly penetrate tumors, it is conceivable that exercise-induced alterations in tumor perfusion may improve delivery of the agent to the tumor. Likewise, it is also possible that regardless of drug delivery, cancer cells in better oxygenated tumors may be less chemoresistant due to decreased expression of drug ABC transporters and anti-apoptotic genes [40]. Further studies are warranted to evaluate the absorption, distribution, metabolism, and excretion (ADME) of doxorubicin in exercising versus non-exercising hosts in both tumors and critical normal tissues.

Lastly, doxorubicin-induced cardiotoxicity was found to be blunted in our exercising mice. Doxorubicin’s cardiotoxicity primarily results from inhibition of topoisomerase 2β activating cell death pathways [41]. Cardiomyocytes experience DNA damage and impaired mitochondrial biogenesis, leading to cardiac remodeling and ultrastructural changes [42, 43]. It is not clear by which mechanism exercise protects against doxorubicin-induced cardiotoxicity. Some studies suggest that exercise upregulates cardiac expression of antioxidant proteins that mitigate ROS damage [44], while others suggest exercise reduces mitochondrial accumulation of doxorubicin by upregulating ABC transporters in cardiomyocytes [45]. Likewise, further ADME studies are needed to analyze how exercise influences the pharmacokinetics and pharmacodynamics of doxorubicin in the hearts of tumor-bearing hosts. It should be noted that this current study was limited by the fact non-tumor bearing hosts were employed in the cardiotoxicity experiment. Still, the observed amelioration of cardiotoxicity by aerobic exercise is very encouraging. Indeed, the strongest correlation between exercise adaptations and tumor-related outcomes was in doxorubicin-induced cardiotoxicity. This suggests that although the amount or intensity of an exercise prescription might not linearly correlate with tumor growth or metastasis outcomes, it may determine cardiac outcomes in a dose-dependent manner.

In oncology, exercise is being considered as an adjuvant to existing anticancer treatments. In the present study, aerobic exercise improved doxorubicin’s anti-tumor efficacy, and also reduced its cardiotoxicity, implying that the inclusion of exercise in a therapeutic regimen led to a therapeutic gain. The phenomena underlying these effects may extend to other treatment modalities, including radiation and immunotherapy [46–48]. Modulation of either arm of the therapeutic index will positively affect patient outcomes, and exercise may uniquely affect both treatment efficacy and toxicity without additional side effects. Future studies will establish if these effects hold true in clinical oncology populations in addition to providing the well-known physical and psychological benefits of exercise [49].

## MATERIALS AND METHODS

### Cell culture

Murine mammary adenocarcinoma cell lines (4T1 and EMT6) were purchased from ATCC (Manassas, VA, USA). 4T1 cells were cultured in RPMI medium supplemented with 10% fetal bovine serum, 1% L-glutamine and 1% penicillin-streptomycin. EMT6 cells were cultured in Waymouth’s medium supplemented with 15% fetal bovine serum, 1% L-glutamine and 1% penicillin-streptomycin. All cells were maintained at 37°C in a humidified atmosphere of 5% CO_2_ and injected *in vivo* between 4–10 passages from freeze. Mycoplasma tests were performed in-house by using MycoAlert Mycoplasma Detection Kit (Walkersville, MD, USA).

### Mouse models of breast cancer and exercise

Female, retired breeder BALB/c mice, 11–12 months of age, were purchased from Envigo (Indianapolis, IN, USA). All experiments were approved by the Institutional Animal Care and Use Committee at the University of Florida. Mice were placed in an induction chamber flooded with 1–3% isoflurane. Upon anesthesia, mice were then placed in dorsal recumbency on an absorbent pad placed over a heated pad (K&H Small Animal Heated Pad, 25 watts) on a previously disinfected stainless-steel table. Fur over the injection site (4th mammary) was removed by depilatory cream. A 32-gauge ½ inch Hamilton syringe, was used to inject 1 × 10^3^ cells, resuspended in 5 μL of sterile Trypan Blue 0.04% in PBS, with the aid of a dissecting microscope for visualization of the injection area, as described previously [50]. Mice were then randomized into non-wheel running (NWR) and exercising (EX) groups. Exercising mice had 24/7 access to low-profile, wireless running wheels purchased from Med Associates Inc. (St. Albans, VT, USA). Individual running distances were recorded via telemetry and binned for 24-hour periods. Non-wheel running mice were housed with stationary plastic huts. All mice were singly housed and were fed *ad libitum* in bowls and given free access to water. Tumor volume was measured via caliper and calculated as (length × width^2^)/2, and endpoint was defined as tumor volume ≥ 1,200 mm^3^. Mice were euthanized via CO_2_ asphyxiation, followed by cervical dislocation and tissues were harvested, weighed, and either snap-frozen in liquid nitrogen or fixed in 10% neutral buffered formalin (lungs were fixed in Bouin’s Solution and macro-metastases were counted using a dissecting microscope). Whole blood was collected and allowed to clot at room temperature for 30 minutes, then centrifuged at 1,500 × g for 10 minutes at 4°C. The supernatant was collected and frozen at −80°C.

### Citrate synthase activity

The activity of the citrate synthase enzyme, a marker for mitochondrial density and oxidative capacity, was measured as described previously [51]. Briefly, snap-frozen quadriceps were crushed via mortar and pestle over dry ice and then homogenized via handheld homogenizer in 100 mM KO_4_; 5 mM EDTA; 5 mM EGTA, pH 7.4. The protein concentration was measured by BCA assay, and 25 μg of total protein was diluted in equal volume. 30 μL of sample were added in duplicate to a 96 well plate. TRIS buffer (100 mM, pH 8.0), DTNB (1 mM), and acetyl coenzyme A (3 mM) were added to each well in 195 μL, 15 μL, and 30 μL volumes, respectively. In a VERSAmax tunable microplate reader from Molecular Devices (San Jose, CA, USA), readings were taken every 30 seconds for 5 minutes at 412 nm at 30°C, and again following addition of oxaloacetate (5mM, 30 μL). The absorptive rate of change was determined for before and after addition of oxaloacetate, and the enzymatic activity was calculated as the difference between the two, presented as nmol/min/μg protein.

### Serum cardiac troponin I ELISA

Circulating levels of cardiac troponin I, a marker for cardiac damage, were determined according to manufacturer instructions of an ELISA kit from Life Diagnostics (West Chester, PA, USA) [52]. Briefly, serum samples were allowed to thaw from −80°C to room temperature. Samples were diluted and added to wells in duplicate. Following incubation and wash steps, absorbance was read at 450 nm and concentrations were determined from standard curve, presented as pg/mL.

### Immunoblot

Snap-frozen tumors were crushed via mortar and pestle over dry ice and then homogenized via handheld homogenizer in RIPA buffer (50 mM Tris–HCl; 150 mM NaCl; 0.1% SDS; 1% NP-40; 0.25% Sodium deoxycholate; 1 mM EDTA, pH 8.0) containing protease inhibitor (Sigma Aldrich), 1 mM NaF, and 1 mM Na_3_VO_4_. The soluble fraction was centrifugally separated, the protein concentration was measured by BCA assay, and 40 μg of protein was diluted in equal volume and boiled in Laemmli loading buffer. Tissue lysates were separated by electrophoresis on SDS-PAGE gels and then transferred to a PVDF membrane. Membrane was blocked for 1 h with 5% BSA in TBS-T (20 mM Tris; 137 mM NaCl; 0.1% Tween-20, pH 7.5), and with a primary antibody diluted in 5% BSA in TBS-T overnight. Membrane was washed for 10 minutes with TBS-T for three times and incubated with secondary antibody diluted in TBS (20 mM Tris, 137 mM NaCl, pH 7.5) for at least 1 h. The signal was detected with an enhanced chemiluminescence substrate (Catalog #: RPN2209, GE Healthcare) and imaged on the Amersham Imager 680 (GE Healthcare). Primary antibodies: HIF-1α (Novus Biologicals, catalog NB100-479SS, dilution 1:1,000), HIF-2α (Novus Biologicals, catalog NB100-122SS, dilution 1:1,000), and β-actin (Sigma Aldrich, catalog A1978, dilution 1:20,000). Horseradish peroxidase-conjugated secondary antibodies: goat anti-mouse IgG (Jackson ImmunoResearch, catalog 115-035-003, dilution 1:20,000), goat anti-rabbit (Jackson ImmunoResearch, catalog 111-035-003, dilution 1:10,000).

### Mass spectrometry metabolomics

Samples were extracted with pre-normalization to the 500 ug/mL sample protein content. Global metabolomics profiling was performed on a Thermo Q-Exactive Oribtrap mass spectrometer with Dionex UHPLC and autosampler. All samples were analyzed in positive and negative heated electrospray ionization with a mass resolution of 35,000 at m/z 200 as separate injections. Separation was achieved on an ACE 18-pfp 100 × 2.1 mm, 2 μm column with mobile phase A as 0.1% formic acid in water and mobile phase B as acetonitrile. The flow rate was 350 μL/minute with a column temperature of 25°C. 4 μL was injected for negative ions and 2 μL for positive ions. MZmine (freeware) was used to identify features, deisotope, align features and perform gap filling to fill in any features that may have been missed in the first alignment algorithm. All adducts and complexes were identified and removed from the data set. MetaboAnalyst 5.0 (https://www.metaboanalyst.ca/) was used to perform sparse partial least squares discriminant analysis of 5 components and 20 variables per component. Hierarchical clustering was performed on the top 50 metabolites as determined by *t*-test.

### Doxorubicin administration

Doxorubicin hydrochloride was purchased from Sun Surgical Supply (Gainesville, FL, USA). This agent was administered via intraperitoneal injection. This agent was delivered in 3 doses over 7 days (on days 0, 3, and 6) for a total dose of either 11 mg/kg or 18 mg/kg. Control mice received equivalent volume of phosphate buffered saline (PBS).

### Statistical analyses

Data are expressed as mean ± SEM or as box-whisker plots (median, interquartile range, range). Welch’s *t*-test, one-way ANOVA, or two-way ANOVA were applied, where appropriate. All statistical analyses were performed using GraphPad Prism software (San Diego, CA, USA). A threshold of *p* < 0.05 was considered significant.

## SUPPLEMENTARY MATERIALS



## Abbreviations

NWR: non-wheel running
EX: exercising
PBS: phosphate-buffered saline
DOXO: doxorubicin
