# Supplementary material for: Normal tissue and tumor microenvironment adaptations to aerobic exercise enhance doxorubicin anti-tumor efficacy and ameliorate its cardiotoxicity in retired breeder mice
Source: Oncotarget. 2021 Aug 31;12(18):1737–48. doi: 10.18632/oncotarget.28057 (PMC8416558; doi:10.18632/oncotarget.28057)
Supplement: Supplementary file 1 [file oncotarget-12-1737-s001.pdf]

Normal tissue and tumor microenvironment adaptations to aerobic exercise enhance doxorubicin anti-tumor efficacy and ameliorate its cardiotoxicity in retired breeder mice

SUPPLEMENTARY MATERIALS

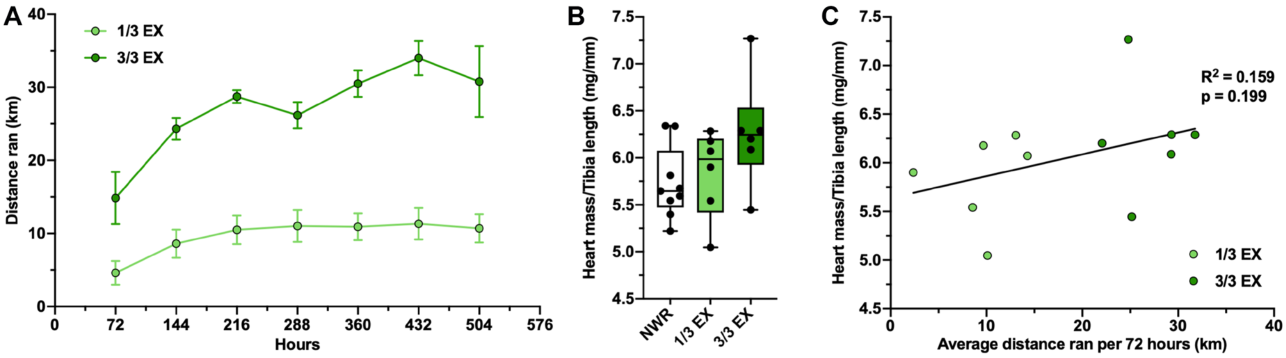

Supplementary Figure 1: Average distance ran per 72-hour period ± SEM in female, retired breeder BALB/c mice when given access to voluntary running wheels 1 day out of every 3 days (1/3 EX) and every day (3/3 EX; (**A**)). Heart mass to tibia length ratio (mg/mm; (**B**)). Linear regression of individual average distance ran per 72 hours and normalized heart mass, Pearson's R^2 values denoted in panel (**C**). One-way ANOVA was used to statistically compare means between groups with Tukey's post-hoc analysis, no statistical significance, study sample size: NWR *n* = 9; 1/3 EX *n* = 6; 3/3 EX *n* = 6.

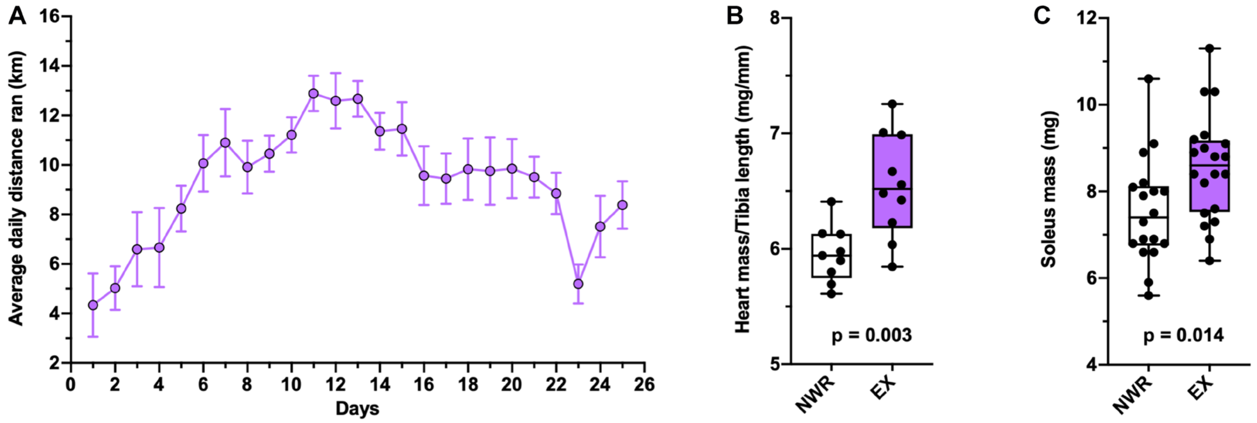

Supplementary Figure 2: Average distance ran per 24-hour period ± SEM in female, retired breeder BALB/c mice with 4T1 intraductal tumors (**A**) Heart mass to tibia length ratio (mg/mm; (**B**)). Soleus mass (mg; (**C**)). Welch's *t*-test was used to statistically compare means between groups, *p* values denoted in panels, study sample size: NWR *n* = 11; EX *n* = 11.

Two-way ANOVA for Figure 5B

Source of Variation	% of total variation	P value	Significant?	
Interaction	0.5705	0.5712	No	
Exercise Status	15.51	0.0051	Yes	
Treatment	20.40	0.0016	Yes	
**Tukey's multiple comparisons test**	**Predicted (LS) mean diff.**	**95.00% CI of diff.**	**Summary**	**Adjusted P Value**
NWR:PBS vs. NWR:DOXO	0.3343	-0.1242 to 0.7928	ns	0.2206
NWR:PBS vs. EX:PBS	-0.4171	-0.8532 to 0.01890	ns	0.0651
NWR:PBS vs. EX:DOXO	0.05144	-0.3948 to 0.4977	ns	0.9895
NWR:DOXO vs. EX:PBS	-0.7514	-1.200 to -0.3029	***	0.0004
NWR:DOXO vs. EX:DOXO	-0.2829	-0.7414 to 0.1757	ns	0.3584
EX:PBS vs. EX:DOXO	0.4686	0.03254 to 0.9046	*	0.0312

Two-way ANOVA for Figure 5E

Source of Variation	% of total variation	P value	Significant?	
Interaction	2.542	0.2474	No	
Exercise Status	4.076	0.1452	No	
Treatment	26.54	0.0005	Yes	
**Tukey's multiple comparisons test**	**Predicted (LS) mean diff.**	**95.00% CI of diff.**	**Summary**	**Adjusted P Value**
NWR:PBS vs. NWR:DOXO	-2.133	-5.307 to 1.041	ns	0.2853
NWR:PBS vs. EX:PBS	-0.2545	-3.273 to 2.764	ns	0.9958
NWR:PBS vs. EX:DOXO	-4.300	-7.389 to -1.211	**	0.0033
NWR:DOXO vs. EX:PBS	1.879	-1.226 to 4.984	ns	0.3753
NWR:DOXO vs. EX:DOXO	-2.167	-5.341 to 1.007	ns	0.2725
EX:PBS vs. EX:DOXO	-4.045	-7.064 to -1.027	**	0.0049

Supplementary Figure 3: Two-way ANOVA results for Figure 5. *p* values are given for main effects, as well as for the interaction between each group.

Two-way ANOVA for Figure 6B

Source of Variation	% of total variation	P value	Significant?	
Interaction	0.02597	0.9069	No	
Exercise status	12.10	0.0163	Yes	
Treatment	31.76	0.0003	Yes	
**Tukey's multiple comparisons test**	**Predicted (LS) mean diff.**	**95.00% CI of diff.**	**Summary**	**Adjusted P Value**
NWR:PBS vs. NWR:DOXO	0.5221	0.04891 to 0.9953	*	0.0262
NWR:PBS vs. EX:PBS	-0.2988	-0.7856 to 0.1881	ns	0.3575
NWR:PBS vs. EX:DOXO	0.1943	-0.2789 to 0.6675	ns	0.6823
NWR:DOXO vs. EX:PBS	-0.8208	-1.294 to -0.3477	***	0.0003
NWR:DOXO vs. EX:DOXO	-0.3278	-0.7868 to 0.1313	ns	0.2327
EX:PBS vs. EX:DOXO	0.4931	0.01988 to 0.9662	*	0.0386

Two-way ANOVA for Figure 6C

Source of Variation	% of total variation	P value	Significant?	
Interaction	4.207	0.3114	No	
Exercise status	0.6257	0.6922	No	
Treatment	33.57	0.0094	Yes	
**Tukey's multiple comparisons test**	**Predicted (LS) mean diff.**	**95.00% CI of diff.**	**Summary**	**Adjusted P Value**
NWR:PBS vs. NWR:DOXO	-854.5	-1719 to 10.22	ns	0.0533
NWR:PBS vs. EX:PBS	-137.3	-1002 to 727.5	ns	0.9679
NWR:PBS vs. EX:DOXO	-544.9	-1410 to 319.8	ns	0.3078
NWR:DOXO vs. EX:PBS	717.2	-147.5 to 1582	ns	0.1229
NWR:DOXO vs. EX:DOXO	309.6	-555.1 to 1174	ns	0.7380
EX:PBS vs. EX:DOXO	-407.7	-1272 to 457.1	ns	0.5471

Two-way ANOVA for Figure 6D

Source of Variation	% of total variation	P value	Significant?	
Interaction	0.4836	0.4974	No	
Exercise status	29.43	<0.0001	Yes	
Treatment	3.976	0.0547	No	
**Tukey's multiple comparisons test**	**Predicted (LS) mean diff.**	**95.00% CI of diff.**	**Summary**	**Adjusted P Value**
NWR:PBS vs. NWR:DOXO	0.2639	-0.5086 to 1.036	ns	0.8043
NWR:PBS vs. EX:PBS	-1.244	-2.039 to -0.4488	***	0.0006
NWR:PBS vs. EX:DOXO	-0.6972	-1.470 to 0.07532	ns	0.0912
NWR:DOXO vs. EX:PBS	-1.508	-2.280 to -0.7351	****	<0.0001
NWR:DOXO vs. EX:DOXO	-0.9611	-1.711 to -0.2116	**	0.0066
EX:PBS vs. EX:DOXO	0.5465	-0.2260 to 1.319	ns	0.2528

Supplementary Figure 4: Two-way ANOVA results for Figure 6. *p* values are given for main effects, as well as for the interaction between each group.

Supplementary Table 1: List of top 50 differentially regulated metabolites by exercise with accompanying fold change (FC) and *p* values from global metabolomics profiling

Metabolite	FC	log2(FC)	*p* value	log10(*p*)
n-_160.0262-0.74	0.53214	–0.91013	0.0051318	2.2897
_137.0456-6.64	1.5137	0.59805	0.011287	1.9474
_154.0474-1.95	1.2438	0.31474	0.020708	1.6839
n-_306.9069-0.62	0.77349	–0.37054	0.023386	1.631
n-_187.0202-0.99	0.66336	–0.59213	0.02853	1.5447
n-_232.9745-0.75	0.77502	–0.3677	0.031562	1.5008
Acyl-Carnitine(5-OH)_262.1642-6.61	1.2593	0.33257	0.035956	1.4442
n-_153.0305-1.51	1.3331	0.41477	0.039314	1.4055
n-_330.9062-0.65	0.88851	–0.17054	0.044368	1.3529
n-_147.0085-0.76	1.1848	0.24464	0.044694	1.3498
_154.0608-6.80	1.2144	0.28028	0.046133	1.336
n-_151.0069-2.00	1.2723	0.3474	0.052253	1.2819
_155.0447-1.51	1.3237	0.40455	0.056292	1.2496
n-N6-(DELTA2-ISOPENTENYL)-ADENINE_202.1084-8.40	1.5566	0.63843	0.057473	1.2405
6-DEOXY-L-GALACTOSE_147.0649-0.84	1.905	0.92979	0.063531	1.197
HOMOCYSTEINE_136.0422-0.91	1.8631	0.89773	0.064538	1.1902
n-_263.0389-7.69	1.2593	0.33266	0.065008	1.187
n-_254.9411-0.63	1.1338	0.1812	0.066335	1.1783
n-_249.1129-11.59	1.2863	0.3632	0.066595	1.1766
n-_73.0289-2.34	1.1589	0.21278	0.068098	1.1669
n-_117.0556-6.08	1.4528	0.53887	0.06917	1.1601
n-_282.9364-0.60	1.1249	0.16982	0.069411	1.1586
_153.0400-6.50	0.64413	–0.63457	0.072457	1.1399
ARABINOSE_133.0494-0.77	1.253	0.32534	0.074501	1.1278
n-_85.0290-2.50	1.264	0.33804	0.076119	1.1185
n-_118.9656-0.60	1.1107	0.15142	0.078515	1.105
n-_250.0234-0.83	2.9208	1.5464	0.079549	1.0994
Isocytosine_112.0507-1.54	0.57498	–0.79841	0.07962	1.099
_132.0654-1.95	1.1986	0.26131	0.080641	1.0934
n-_291.0832-1.44	1.2628	0.33664	0.081899	1.0867
_119.0163-0.91	1.5606	0.64213	0.085495	1.0681
_136.0614-5.75	0.86266	–0.21314	0.087302	1.059
n-D-SACCHARIC ACID_209.0299-0.74	0.81261	–0.29937	0.087538	1.0578
n-_281.0890-6.68	1.4364	0.52247	0.087707	1.057
n-_299.0786-8.05	1.3662	0.45015	0.088131	1.0549
n-_223.1340-11.57	0.75624	–0.40309	0.08824	1.0543
n-Sulfoacetaldehyde_122.9759-0.71	0.86845	–0.20348	0.091263	1.0397
n-2-deoxy-D-galactose (fructose/glucose)_163.0612-0.93	1.2321	0.30117	0.091294	1.0396
n-6-DEOXY-L-GALACTOSE_163.0611-0.85	1.2321	0.30117	0.091294	1.0396
n-_299.0786-8.24	1.3728	0.4571	0.092516	1.0338
N-ACETYLGLYCINE_118.0501-1.23	1.2381	0.30808	0.093197	1.0306
n-2-DEOXY-D-GLUCOSE_209.0665-0.81	1.3072	0.38647	0.093358	1.0298
_131.0813-1.12	1.2909	0.3684	0.09417	1.0261
_143.0812-1.01	1.2623	0.336	0.095098	1.0218
L-KYNURENINE_209.0917-6.41	0.48137	–1.0548	0.095119	1.0217
n-5′-DEOXYADENOSINE_296.0985-6.70	1.4944	0.57961	0.095794	1.0187
CITRATE_193.0340-1.94	1.3208	0.40146	0.09683	1.014
n-_202.0334-6.12	1.432	0.51799	0.097934	1.0091
n-_311.2228-12.60	0.42493	–1.2347	0.098932	1.0047
_158.1172-1.81	1.2234	0.29085	0.099063	1.0041
